# Non-invasive respiratory support for infants with bronchiolitis: a national survey of practice

**DOI:** 10.1186/s12887-017-0785-0

**Published:** 2017-01-17

**Authors:** H. Turnham, R. S. Agbeko, J. Furness, J. Pappachan, A. G. Sutcliffe, P. Ramnarayan

**Affiliations:** 1 Bristol Royal Hospital for Children, University Hospitals Bristol NHS Foundation Trust, Bristol, United Kingdom; 2 Great North Children’s Hospital, The Newcastle upon Tyne Hospitals NHS Foundation Trust, Newcastle University, Newcastle upon Tyne, UK; 3 Institute of Cellular Medicine, Newcastle University, Newcastle upon Tyne, UK; 4 Country Durham and Darlington NHS Foundation trust, Darlington, UK; 5 Southampton Children’s Hospital, Southampton, UK; 6 Institute of Child Health, University College London, GAP unit, Institute of Child Health, 30 Guilford Street, London, WC1N 1EH UK; 7 Children’s Acute Transport Service, Great Ormond Street Hospital NHS Foundation Trust, London, UK

**Keywords:** Bronchiolitis, Respiratory failure, Non-invasive respiratory support, Critical care

## Abstract

**Background:**

Bronchiolitis is a common respiratory illness of early childhood. For most children it is a mild self-limiting disease but a small number of children develop respiratory failure. Nasal continuous positive airway pressure (nCPAP) has traditionally been used to provide non-invasive respiratory support in these children, but there is little clinical trial evidence to support its use. More recently, high-flow nasal cannula therapy (HFNC) has emerged as a novel respiratory support modality. Our study aims to describe current national practice and clinician preferences relating to use of non-invasive respiratory support (nCPAP and HFNC) in the management of infants (<12 months old) with acute bronchiolitis.

**Methods:**

We performed a cross-sectional web-based survey of hospitals with inpatient paediatric facilities in England and Wales. Responses were elicited from one senior doctor and one senior nurse at each hospital. We analysed the proportion of hospitals using HFNC and nCPAP; clinical thresholds for their initiation; and clinician preferences regarding first-line support modality and future research.

**Results:**

The survey was distributed to 117 of 171 eligible hospitals; 97 hospitals provided responses (response rate: 83%). The majority of hospitals were able to provide nCPAP (89/97, 91.7%) or HFNC (71/97, 73.2%); both were available at 65 hospitals (67%). nCPAP was more likely to be delivered in a ward setting in a general hospital, and in a high dependency setting in a tertiary centre. There were differences in the oxygenation and acidosis thresholds, and clinical triggers such as recurrent apnoeas or work of breathing that influenced clinical decisions, regarding when to start nCPAP or HFNC. More individual respondents with access to both modalities (74/106, 69.8%) would choose HFNC over nCPAP as their first-line treatment option in a deteriorating child with bronchiolitis.

**Conclusions:**

Despite lack of randomised trial evidence, nCPAP and HFNC are commonly used in British hospitals to support infants with acute bronchiolitis. HFNC appears to be currently the preferred first-line modality for non-invasive respiratory support due to perceived ease of use.

**Electronic supplementary material:**

The online version of this article (doi:10.1186/s12887-017-0785-0) contains supplementary material, which is available to authorized users.

## Background

Bronchiolitis is a common respiratory illness of young childhood caused by viruses such as Respiratory Syncytial Virus (RSV) [1]. Bronchiolitis is usually a mild, self-limiting disease, but 2–5% of children require hospitalisation [1–6], while 1–2.7% need critical care support [7, 8].

Nasal continuous positive airway pressure (nCPAP) has been used as a mode of non-invasive respiratory support for infants with bronchiolitis-induced respiratory failure for over two decades [9–13], and is increasingly being used in a ward setting [14]. Recently, high-flow nasal cannula therapy (HFNC) has become a popular alternative [15]. However, investment and training in new equipment is expensive, and concerns persist regarding the safety of HFNC (risk of pneumothorax or pneumomediastinum and nosocomial infection) and the potential for delay in initiating invasive ventilation for high-risk children [16–20].

A previous survey of UK neonatal units demonstrated widespread adoption of HFNC despite the absence of robust clinical trial evidence [21]. We aimed to establish current national practice in the management of infants with acute bronchiolitis by conducting a cross-sectional survey of clinicians working in hospitals in England and Wales. We also aimed to determine clinician preferences regarding clinical triggers to initiate nCPAP and HFNC, and to ascertain if clinical equipoise existed to support a multicentre trial of non-invasive respiratory support in acute bronchiolitis.

## Methods

### Survey

We designed a cross-sectional survey of hospitals with inpatient paediatric facilities. An online questionnaire using Survey Monkey software (www.surveymonkey.com, Survey Monkey, Palo Alto, USA) was piloted by three senior doctors from different parts of England (authors JP, AS and JF). Feedback from the pilot was used to finalise the questionnaire used in the study. The final questionnaire covered three main themes: availability and use of nCPAP and HFNC at hospital level, clinical criteria for the initiation of nCPAP and HFNC, and clinician preferences towards future research. The final questionnaire is available as Additional file 1. Survey responses were elicited using two methods: a) a survey link emailed to the lead consultant and lead nurse in all 25 tertiary paediatric hospitals and b) a survey link sent to each of the 12 regional paediatric intensive care retrieval services, who were asked to forward the questionnaire to the designated consultant and senior nurse at each of the district general hospitals in their regional network. Completion of the survey was voluntary and consent was implied though completion and submission of the survey. The initial survey distribution was followed up by reminder emails 4 and 8 weeks later. Survey data were collected between June and October 2014.

Data collected by the Royal College of Paediatrics and Child Health for the Medical Workforce Census 2013 was used to provide data regarding the number and size of all hospitals admitting children as in-patients in the United Kingdom [22]. We used the definition of High Dependency Care (HDC) as that defined in England and Wales by the Royal College of Paediatrics and Child Health: ‘care for a child who requires enhanced observation, monitoring or intervention than cannot be delivered on a standard paediatric ward’ [23]. The Royal College of Nursing have published recommendations for minimum nurse staffing levels for general paediatric wards (1 nurse per 3 infants), high dependency units (1 nurse per 2 patients) and intensive care units (1 nurse per patient) [24].

### Data analysis

We used the hospital as the unit of analysis for questions relating to hospital practice. If discrepancies were identified between respondents from the same hospital, we chose to use the senior doctor’s response for this analysis. We used the respondent as the unit of analysis for questions relating to clinical thresholds and beliefs regarding clinical equipoise. Results are reported as proportions and/or means as appropriate. Significance testing for differences in proportions were performed using the chi-square test, and for differences in means for normally distributed data using the Student *t*-test. We performed multivariate analysis to study the association between hospital type (tertiary vs. general hospital) and the use of HFNC and/or nCPAP, respondent type (consultant vs. nurse), preference of first-line modality and willingness to participate in future research, using the survey statistics module of Stata IC v13 (Stata Corporation, College Station, USA). The hospital was set as the primary sampling unit, and accounted for clustering of respondents within each hospital. Data analyses were performed using Stata and Microsoft Excel (Microsoft Corporation, USA).

## Results

The survey was disseminated to 117 out of 171 eligible hospitals; responses were obtained from 97 hospitals (response rate: 83%). Survey responses were received from all 25 tertiary hospitals. The survey was distributed by regional PICU retrieval services to 8 out of 12 regions in England and Wales, reaching 92 general hospitals, of which 72 responded. Survey responses were provided by 159 individual respondents (mean: 1.6 responses per hospital). The majority of respondents were paediatric consultants (102/159, 64.2%) and the remainder were senior nurses.

Characteristics of the hospitals from which responses were obtained are shown in Table 1.

**Table 1:** Characteristics of hospitals who responded to the survey (*n* = 97)

	Tertiary centre *n* = 25 (%)	General hospital *n* = 72 (%)
Hospital size^a^		
Very small (≤1500 admissions per year)	1 (4.0)	2 (2.8)
Small (1501–2500 admissions per year)	4 (16.0)	14 (19.4)
Medium (1501–5000 admissions per year)	9 (36.0)	34 (47.2)
Large (5001–6000 admissions per year)	3 (12.0)	9 (12.5)
Very large (>6000 admissions per year)	8 (32.0)	13 (18.1)
Care areas in hospital		
Dedicated paediatric high dependency unit	22 (88.0)	9 (12.5)
High dependency beds within paediatric ward	4 (16.0)	41 (56.9)
No paediatric high dependency beds	3 (12.0)	23 (31.9)
Dedicated paediatric intensive care beds	23 (92.0)	0 (0)
Dedicated paediatric emergency department	18 (72.0)	12 (16.7)
Bronchiolitis guideline used		
Local	20 (80.0)	62 (86.1)
Regional	3 (12.0)	13 (18.1)
National	2 (8.0)	13 (18.1)
No guideline	2 (8.0)	3 (4.2)
Availability of non-invasive respiratory support		
nCPAP	24 (96.0)	65 (90.3)
HFNC	20 (80.0)	51 (70.8)
Either HFNC or nCPAP	25 (100)	70 (97.2)
Both	19 (76.0)	46 (63.9)

^a^Classification based on number of paediatric inpatient admissions per year (as per the RCPCH Medical Workforce Census 2013)

### Availability of paediatric high dependency units (PHDU)

Most tertiary hospitals (88%) reported that they had a dedicated PHDU compared to only 12.5% of general hospitals, where children requiring high dependency level care were more likely to be cared for in dedicated PHDU beds located within the general ward (57%). 26 general hospitals (26.8%) reported no availability of PHDU.

### Guidelines and protocols

No guidelines were used by 5 hospitals for the management of bronchiolitis. The remainder used local, regional and/or national guidelines. Overall, general hospitals were more likely to use guidelines than tertiary hospitals. The majority of hospitals had local guidelines in place (82/97, 84.5%).

### Hospital practice regarding non-invasive respiratory support in acute bronchiolitis

#### nCPAP

Twenty-four (96%) tertiary hospitals and 65 (90%) general hospitals reported being able to provide nCPAP. Of the 8 hospitals that did not use nCPAP, one commented that there was a lack of evidence to support its use and another that absence of adequate staff training prevented its use. nCPAP was delivered in a ward setting by 4 out of 25 (16%) tertiary hospitals and 41 out of 72 (56.9%) general hospitals, whereas it was more likely to be delivered in a PHDU or PICU setting in a tertiary centre.

Use of sedation to facilitate the provision of nCPAP was elicited from individual responders by asking them how often sedation was used: always, sometimes, rarely or never. Use of sedation was variable: 41/97 hospitals used sedation sometimes or routinely (42.3%) while 37/97 (38.1%) used it rarely. Nine hospitals reported never using sedation, while 9 hospitals did not submit information. Tertiary hospitals were more likely to use sedation sometimes or routinely than general hospitals (18/25, 72% versus 24/72, 33.3%, *p* = 0.003).

#### HFNC

Twenty (80%) of tertiary hospitals and 51 (70.8%) of general hospitals reported being able to provide HFNC (a further 9 hospitals were planning to implement the technology within the next 12 months). HFNC was delivered more frequently in the ward setting in general hospitals (38/72, 52.8% vs. 6/25, 24%), whereas it was more likely to be delivered in a HDU or PICU setting in a tertiary centre (20/25, 80% vs. 20/72, 27.8%).

We asked responders about hospital guidelines for maximal flow rates for HFNC in particular areas of their hospital. We used this as a reflection of safety concerns regarding introduction of this new technology. Not all responders commented, however, there is a trend towards higher flow rates being tolerated on paediatric wards and high dependency areas than in emergency departments of both tertiary and general hospitals (Table 2).

**Table 2:** Maximal flow rates used locally for HFNC in tertiary and general hospitals

	Tertiary Hospitals (*n* = 20)			General Hospitals (*n* = 51)		
	1–5 L/min	6–10 L/min	>10 L/min	1–5 L/min	6–10 L/min	>10 L/min
Emergency Department	0	2 (10%)	0	2 (3%)	4 (7.8%)	7 (14%)
Paediatric Ward	0	5 (25%)	3 (15%)	3 (5.8%)	18 (35%)	18 (35%)
Paediatric High Dependency Ward	1 (5%)	6 (30%)	6 (30%)	5 (10%)	18 (35%)	13 (25%)

Either nCPAP or HFNC was available in all tertiary hospitals and in nearly all general hospitals (70/72, 97.2%), while two-thirds of the hospitals had access to both modalities (65/97, 67%).

### Individual clinicians’ practice regarding non-invasive respiratory support in acute bronchiolitis

**Fig. 1:**
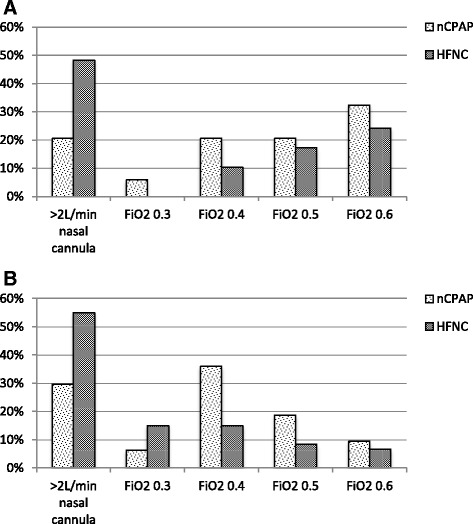
Oxygen requirement threshold at which clinicians would start HFNC/nCPAP at tertiary hospitals (panel **a**) and general hospitals (panel **b**). Graphs show a breakdown of available responses: panel **a** – 34 (NCPAP) and 29 (HFNC) responses from 50 clinicians; panel **b** – 64 (nCPAP) and 60 (HFNC) responses from 109 clinicians

**Fig. 2:**
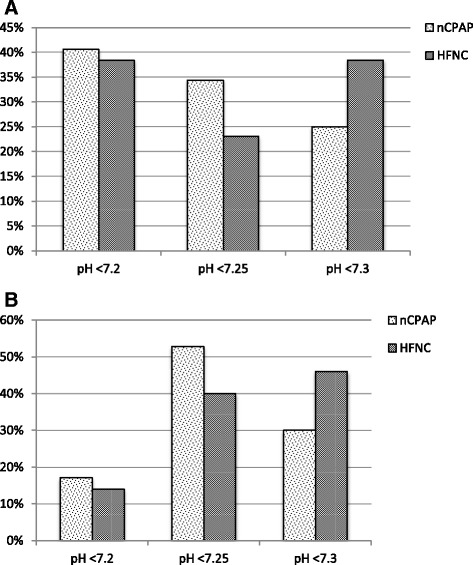
Acidosis threshold at which clinicians would start nCPAP/HFNC at tertiary hospitals (panel **a**) and general hospitals (panel **b**). Graphs show a breakdown of available responses: panel **a** – 32 (NCPAP) and 26 (HFNC) responses from 50 clinicians; panel **b** – 70 (nCPAP) and 50 (HFNC) responses from 109 clinicians

Figure 1 illustrates oxygen requirement criteria that clinicians currently use to initiate nCPAP and HFNC in infants with acute bronchiolitis in tertiary hospitals (panel A) and general hospitals (panel B). Fig. 2 illustrates acidosis criteria that clinicians currently use to initiate nCPAP and HFNC in infants with acute bronchiolitis in tertiary hospitals (panel A) and general hospitals (panel B). A significant number of clinicians (see legends of Figs. 1 and 2) did not respond to these two questions, citing that they would not use specific oxygenation and acidosis criteria in isolation.

**Fig. 3:**
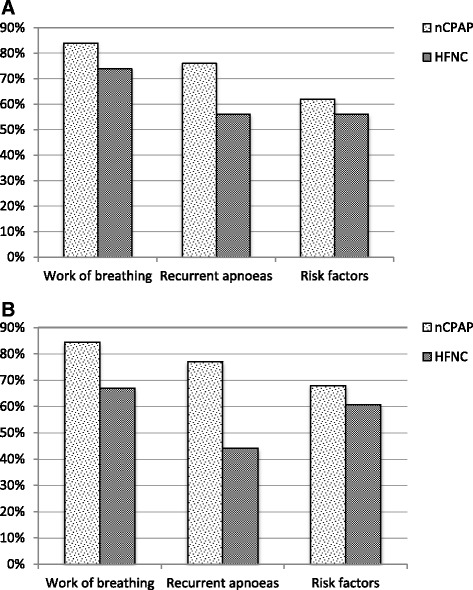
Clinical factors that influence decision to start nCPAP/HFNC at tertiary hospitals (panel **a**) and general hospitals (panel **b**)

Figure 3 illustrates the clinical criteria of work of breathing, recurrent apnoeas and presence of high-risk co-morbid conditions (e.g., prematurity or cardiac disease) that influence the decision to initiate nCPAP and HFNC in tertiary hospitals (panel A) and general hospitals (panel B).

### Clinician preferences for first-line modality

In response to a clinical vignette describing a 6-month old infant with acute bronchiolitis and respiratory distress, the majority of clinicians who had access to both nCPAP and HFNC (74/106, 69.8%) reported that they would start HFNC as the first-line treatment rather than nCPAP. When asked what they perceived the role of HFNC to be in clinical practice, many reported that they considered it as an alternative to nCPAP (78/106, 73.5%) or as a step up before nCPAP (84/106, 79.2%). A smaller proportion felt that it was also useful as a step-down therapy after discontinuation of nCPAP (63/106, 59.4%). There were no significant differences between tertiary hospitals and general hospitals in terms of clinician preference for first-line support mode.

### Future research

When asked to rate the importance of various outcome measures to study in future research on a Likert scale (1: least important; 5: most important), clinicians rated reduction in the need for intubation and ventilation (mean score: 4.8 for general hospital respondents and 4.5 for tertiary centre respondents, *p* = 0.01) and avoiding transfer to another hospital (mean score: 4.7 for general hospitals and 4.0 for tertiary hospitals, *p* < 0.001) as the most important (Table 3). Half of all clinicians who responded were prepared to randomise children with acute bronchiolitis to either nCPAP or HFNC in a future clinical trial (80/159, 50.3%). An additional 42 clinicians (26.4%) would consider participation in an RCT, subject to the study design (free text comments indicated that the ability to crossover between treatment arms was an important consideration). A small proportion (9/159, 5.6%) reported that they were unwilling to participate in a trial due to their belief in the superiority of HFNC compared to nCPAP.

**Table 3:** Patient outcomes viewed by clinicians as being important for study in future research (reported as a score, 1 indicating least important, 5 indicating very important)

	Tertiary hospitals (*n* = 50) Mean (SD) score	General hospitals (*n* = 109) Mean (SD) score
Reduction of rate of intubation and ventilation	4.5 (1.0)	4.8 (0.7)
Reduction in need for inter-hospital transfer	4.0 (1.2)	4.7 (0.7)
Reduction in length of stay	3.9 (1.0)	4.2 (0.8)
Reduction in complication rate	3.9 (1.2)	4.3 (0.9)
Improved patient tolerance	3.9 (1.1)	4.2 (0.9)
Reduced need for sedation	3.6 (1.2)	3.5 (1.2)
Parent/Carer Satisfaction	4.0 (0.9)	3.9 (1.1)

### Multivariate analysis

Hospital type was not associated with the availability of HFNC either on its own, or availability of both support modalities, when adjusted for hospital size (*p* = 0.28 and *p* = 0.17 respectively). Respondent type was not associated with choosing HFNC as first-line treatment or with willingness to participate in a future trial, when adjusted for hospital type and size (*p* = 0.65 and *p* = 0.89 respectively).

### Generalisability

In order to assess the generalisability of our findings, we compared the 54 general hospitals that were not surveyed with the 72 hospitals that were surveyed – there was no significant difference in the hospital size between the two groups (*p* = 0.51). Similarly, we compared the 72 general hospitals that responded to the survey with the 25 hospitals that did not respond – there was no significant difference in the hospital size (*p* = 0.53).

## Discussion

Our national survey of hospitals reveals that the use of nCPAP and HFNC is widespread in young children with bronchiolitis. nCPAP appears to be used more frequently in high dependency and intensive care areas, while HFNC use is more frequent in paediatric wards. Clinicians appear to view HFNC and nCPAP as interchangeable modalities, but HFNC appears to be their preferred first-line support option.

nCPAP has been the traditional modality of respiratory support for bronchiolitis for over two decades [9]. It may help to maintain patency of small bronchioles, improve secretion clearance, gas exchange and reduce work of breathing [25]. Although small studies suggest a trend towards physiological improvement with early nCPAP use [12, 26], its impact on outcomes such as length of hospital stay and need for intubation and invasive ventilation have yet to be confirmed in large randomised controlled trials [27]. More recently, HFNC has increased in popularity [15]. HFNC delivers a gas mixture of oxygen/air, warmed to 34–37° Celsius with a relative humidity of almost 100%, at high flow rates. It reduces airway resistance, washes out end-expiratory gases and provides positive airway pressure, reducing work of breathing and improving in gas exchange [28–30]. It is also well tolerated [31].

Our survey findings are similar to those of a recent survey of UK neonatal units −77% of neonatal units are using HFNC, mainly as an alternative to nCPAP [21]. Similar findings have been reported from Australia and New Zealand [32]. However, concerns regarding the safety of HFNC, and reports that it may delay timely access to invasive ventilation, do not support widespread adoption without ensuring an adequate level of clinical monitoring [17, 18].

Our survey results are important for several reasons. First, this is the first national survey of current practice in paediatrics relating to the use of non-invasive support for acute bronchiolitis. Both nCPAP and HFNC are available at most hospitals, but their use is variable and the clinical thresholds at which they are initiated are often different. Second, despite limited evidence, we have shown that HFNC appears to be the current preferred first-line support modality for infants with bronchiolitis. Third, despite enthusiasm for the use of HFNC, the majority of respondents were in clinical equipoise and were willing to participate in a future clinical trial, but a small proportion were not, a number that is only likely to rise in the face of increasing use and the absence of forthcoming evidence. Future studies should focus on clinical outcomes such as reduction in the need for intubation and ventilation and/or need for inter-hospital transfer.

Our survey had several strengths and limitations. We chose for practical purposes to send the survey link first to the regional retrieval services for onward dissemination, rather than directing it to each individual hospital. Even though the survey link was sent to all 12 PICU retrieval services, only 8 disseminated the survey to their network hospitals, thereby resulting in lower coverage than anticipated (54% of hospitals with inpatient paediatric facilities). However, since this was not a systematic process, it is unlikely to have resulted in significant bias. Indeed, we showed that hospitals that were not surveyed were similar to the ones that were surveyed, and that responders were similar to non-responders. The high survey response rate (83%) allows firm conclusions to be drawn regarding current practice. It is also worth highlighting that this was a self-reported questionnaire and as such, may not reflect actual practice, for which an audit of practice may be more useful. We also acknowledge that we studied a rapidly evolving field where clinical practice may already have changed since the survey was conducted.

## Conclusions

Despite the paucity of supportive evidence, nCPAP and HFNC are routinely used to support infants with acute bronchiolitis. HFNC appears to be the preferred first-line modality although the indications for its use and clinical thresholds for its initiation are variable. There remains sufficient equipoise among clinicians to support a national randomised trial of non-invasive respiratory support in acute bronchiolitis.

## Additional file

Additional file 1: Survey questionnaire. (PDF 251 kb)

## Abbreviations

GH: General Hospital
HFNC: High Flow Nasal Cannula
nCPAP: Nasal continuous positive airway pressure
PHDU: Paediatric high dependency unit
PICU: Paediatric intensive care unit
RCT: Randomised controlled trial
RSV: Respiratory syncytial virus
TC: Tertiary centre
