# Supplementary material for: Non-invasive respiratory support for infants with bronchiolitis: a national survey of practice
Source: BMC Pediatr. 2017 Jan 17;17:20. doi: 10.1186/s12887-017-0785-0 (PMC5240267; doi:10.1186/s12887-017-0785-0)
Supplement: Additional file 1: — Survey questionnaire. (PDF 251 kb) [file 12887_2017_785_MOESM1_ESM.pdf]

1. Introduction

The literature suggests that 3-6% of children admitted to hospital with acute bronchiolitis deteriorate and require respiratory support. Traditionally nasal continuous positive airway pressure (nCPAP) has been used as the preferred modality for non-invasive respiratory support in this setting. Children who fail nCPAP usually require intubation and invasive ventilation.

Heated and humidified High Flow Nasal Cannula (HFNC) therapy has emerged as an alternate mode of non-invasive respiratory support over the past decade, particularly in neonatal practice. It is also being increasingly used in infants and children with a variety of conditions. Observational studies suggest that HFNC is better tolerated compared to nCPAP, requires less nursing input, and may reduce the need for intubation and invasive ventilation in children with bronchiolitis. If this is confirmed in a randomised trial, it would have a significant impact on the demand for PICU beds, particularly over winter.

This national survey aims to clarify the current use of non-invasive respiratory support. In particular, we are interested in how, when, and what respiratory support modalities (nCPAP and/or HFNC) are used in hospitals across the country in young children (under 24 months) with bronchiolitis. The findings from this survey are crucial and will help to plan for a multi-center, prospective trial of respiratory support in bronchiolitis.

Thank you very much for your help with this survey.

2. Demographics

Please tell us a little bit about yourself and the hospital/area that you currently work in

1. What departments and care area's are available in your hospital? Tick all that apply

- ☐ General paediatric beds
- ☐ General paediatric ward with dedicated HDU beds
- ☐ Dedicated paediatric HDU
- ☐ Dedicated pediatric intensive care
- ☐ Dedicated paediatric emergency department

Other (please specify)

2. What is the name of your hospital?

3. In which area do you normally work?

- ◯ General Paediatrics
- ◯ Emergency medicine
- ◯ Paediatric emergency medicine
- ◯ General Paediatrics with respiratory interest
- ◯ General Paediatrics with high dependency interest
- ◯ Paediatric Intensive Care
- ◯ Adult intensive care
- ◯ Paediatric Anaesthesia
- ◯ Adult anaesthesia with paediatric interest
- ◯ Adult anaesthesia

Other (please specify)

4. What is your position?

- ◯ Consultant
- ◯ Speciality Trainee ST 4-8/Specialist Registrar/Equivalent
- ◯ Speciality Trainee ST1-3
- ◯ Ward Sister/ Charge Nurse
- ◯ Ward Nurse
- ◯ Respiratory Specialist Nurse
- ◯ Nurse Practitioner

Other (please specify)

3. Treatment of children <24 months with a clinical diagnosis of bronchiolitis in your hospital

Please tell us about your current practice, especially regarding the use of non-invasive respiratory support modalities (nCPAP and/or HFNC) in bronchiolitis

1. Do you use a guideline or protocol to manage children with bronchiolitis in your hospital, tick all that apply

- ☐ Local guideline
- ☐ Regional guideline
- ☐ NICE guideline
- ☐ No guideline

Other (please describe)

* 2. Do you currently manage children requiring non invasive respiratory support (nCPAP and/or HFNC) in your hospital?

- ○ Always
- ○ Usually
- ○ Sometimes
- ○ Rarely
- ○ Never

4.

1. Why don't you currently use non-invasive respiratory support for bronchiolitis in your hospital?

- [ ] No evidence base for non invasive respiratory support
- [ ] Concern over complications
- [ ] Devices not available
- [ ] No trained staff

Other (please specify)

2. Do you have plans to introduce non invasive respiratory support in your hospital?

- ( ) nCPAP
- ( ) HFNC
- ( ) Both
- ( ) Neither

Other (please specify)

5. Non Invasive Continuous Positive Airway Pressure Ventilation (nasal CPAP)

This section looks at the use of nCPAP in bronchiolitis

* 1. Can you deliver nCPAP to children with bronchiolitis in your hospital?

- ○ No
- ○ Yes

Comments

6.

1. If you cannot deliver nCPAP currently are there plans to introduce it for children with bronchiolitis in your hospital in the next 12 months?

- Yes
- No

Other (please specify)

7.

1. In which areas are you able to deliver nCPAP to children with bronchiolitis in your hospital? Tick all that apply

- [ ] Paediatric assesement/ Short stay ward
- [ ] General Paediatric ward
- [ ] Speciality respiratory pediatric ward
- [ ] Paediatric high dependency ward
- [ ] Paediatric intensive care
- [ ] Accident and Emergency with specialist paediatric emergency department
- [ ] Accident and Emergency without specialist paediatric emergency department
- [ ] Adult high dependency
- [ ] Adult intensive care unit

Other (please specify)

2. What do you use to deliver nCPAP to children with bronchiolitis in your hospital?

- [ ] Fabian CPAP
- [ ] Infant flow driver
- [ ] Infant flow SiPAP
- [ ] Bubble CPAP

Other (please specify)

* 3. When would you initiate nCPAP in children with bronchiolitis receiving supplemental oxygen in your hospital?

- ◯ SaO2 < 92% in > 2L/min nasal cannula oxygen
- ◯ SaO2 < 92% in FiO2 0.3
- ◯ SaO2 < 92% in FiO2 0.4
- ◯ SaO2 < 92% in FiO2 0.5
- ◯ SaO2 < 92% in FiO2 0.6
- ◯ Other

Comment

[ ]

* 4. What level of acidaemia would trigger initiation of nCPAP in your hospital in children with bronchiolitis?

- ◯ Acidaemia (pH < 7.3)
- ◯ Acidaemia (pH < 7.25)
- ◯ Acidaemia (pH < 7.2)
- ◯ Other

Comment

[ ]

5. What other indications might trigger the decision to initiate nCPAP in your hospital in children with bronchiolitis?

- ☐ Increased work of breathing (costal recession, tracheal tug, grunting)
- ☐ Infant with known risk factors (eg chronic lung disease)
- ☐ Recurrent apnoeas

Other (please specify)

[ ]

* 6. Do you use sedation to facilitate nCPAP in children with bronchiolitis in your hospital?

- ◯ Always
- ◯ Usually
- ◯ Sometimes
- ◯ Rarely
- ◯ Never

8. High Flow Nasal Cannula Therapy (HFNC)

This section looks at the use of HFNC

* 1. Are you able to deliver HFNC to children with bronchiolitis in your hospital?

- ( ) Yes
- ( ) No

9.

1. Are there plans to introduce HFNC therapy to your hospital to treat children with bronchiolitis in the next 12 months?

- ◯ Yes
- ◯ No

Comment

10.

1. In your hospital, how do you use HFNC? Tick all that apply

- [ ] As an alternative to nCPAP
- [ ] To escalate therapy from low flow oxygen, before starting nCPAP
- [ ] As a method of weaning from nCPAP

Additional comments:

2. In which areas are you able to deliver HFNC to children with bronchiolitis in your hospital? Tick all that apply

- [ ] Paediatric assesement/ Short stay
- [ ] Paediatric general ward
- [ ] Paediatric specialist respiratory ward
- [ ] Paediatric high dependency ward
- [ ] Paediatric intensive Care
- [ ] Accident and emergency with specialist paediatric department
- [ ] Accident and emergency without speciality paediatric department
- [ ] Adult high dependency ward
- [ ] Adult intensive care
- [ ] Other (please specify)

3. What do you use to deliver HFNC in your hospital for children with bronchiolitis?

- ○ Vapotherm
- ○ Optiflow
- ○ Airvo

Other (please specify)

* 4. When would you initiate HFNC in children with bronchiolitis receiving supplemental oxygen in your hospital?

- ◯ SaO2 < 92% in > 2L/min nasal canulae
- ◯ SaO2 < 92% in FiO2 0.3
- ◯ SaO2 < 92% in FiO2 0.4
- ◯ SaO2 < 92% in FiO2 0.5
- ◯ SaO2 < 92% in FiO2 0.6
- ◯ Other

Comment

* 5. What level of acidaemia would trigger initiation of HFNC in your hospital in children with bronchiolitis?

- ◯ Acidaemia (pH < 7.3)
- ◯ Acidaemia (pH < 7.25)
- ◯ Acidaemia (pH < 7.2)
- ◯ Other

Comment

* 6. What other indications might trigger the decision to initiate HFNC in your hospital in children with bronchiolitis?

- ☐ Increased work of breathing
- ☐ Infant with known risk factors (eg chronic lung disease)
- ☐ Recurrent apnoea's
- ☐ Other

Comment

11. Please answer the following question according to the clinical vignette below.

A 6 month old infant is admitted to the children's ward with respiratory distress. She has a three day history of poor feeding and increased work of breathing. The senior nurse reviews the child and finds that she has a respiratory rate of 60 per min, moderately severe sub costal recession and tracheal tug, oxygen saturations of 92% in 2L nasal cannulae oxygen and a heart rate of 180 min. You decide to try HFNC

* 1. What is the maximal flow rate that you are able to deliver to this child in the following three clinical area's in your hospital?

	Not Applicable	Cannot deliver HFNC	1-5 L/min	6-10 L/min	> 10 L/min
Accident and Emergency	☐	☐	☐	☐	☐
Paediatric ward	☐	☐	☐	☐	☐
Paediatric high dependency	☐	☐	☐	☐	☐

12. Please answer the following question according to the clinical vignette below

A 6 month old infant is admitted to the children's ward with respiratory distress. She has a three day history of poor feeding and increased work of breathing. The senior nurse reviews the child and finds that she has a respiratory rate of 70 min, moderately severe sub costal recession and tracheal tug, oxygen saturations of 92% in 2L nasal canulae oxygen and a heart rate of 180 min.

* 1. You decide the baby needs respiratory support, what would you use first?

- [ ] High Flow Nasal Cannula (HFNC)
- [ ] Nasal non-invasive continuous positive pressure ventilation (nCPAP)
- [ ] Other

Comment

[ ]

13. Future Research

In this section, please tell us your views about future research on non-invasive respiratory support modalities in young children with bronchiolitis

* 1. If there was a trial of HFNC as a modality of non-invasive respiratory support, what outcome measures would you consider important? Score importance of each from 1-5 (1: least important, 5: very important)

	1	2	3	4	5
Reduce the need for intubation and invasive ventilation	○	○	○	○	○
Reduced need for transfer to higher level of care	○	○	○	○	○
Reduced length of hospital stay	○	○	○	○	○
Reduced complications (eg pneumothorax, nasal pressure sores)	○	○	○	○	○
Improved tolerance of respiratory support	○	○	○	○	○
Reduced need for sedation	○	○	○	○	○
Improved parent/ carer satisfaction	○	○	○	○	○

Other (please specify)

2. In a multi-centre, prospective, randomised controlled trial comparing HFNC to nCPAP in children with bronchiolitis would you be prepared to randomise children to either modality?

- ○ Yes
- ○ No
- ○ Perhaps

Please provide additional comments, especially if you said Perhaps

3. Please insert the name and contact details of an individual who could be contacted for further discussions about any future trial

4. If you are willing to be contacted about the survey please insert your email address
